# Characteristics of the vertical variation in water quality indicators of aquatic landscapes in urban parks: A case study of Xinxiang, China

**DOI:** 10.1371/journal.pone.0314860

**Published:** 2024-12-05

**Authors:** Yichuan Zhang, Wenke Qin, Lifang Qiao

**Affiliations:** 1 School of Horticulture and Landscape Architecture, Henan Institute of Science and Technology, Xinxiang, China; 2 Henan Province Engineering Center of Horticultural Plant Resource Utilization and Germplasm Enhancement, Xinxiang, China; Makerere University College of Natural Sciences, UGANDA

## Abstract

The quality of landscape water directly impacts the recreational and leisure experiences of the public. Factors such as water clarity, color, and taste can influence public perception, while contaminants like heavy metals, algae, and microorganisms may pose health risks. Stratified monitoring can reveal variations in the physical, chemical, and biological properties of water at different depths, thereby providing a more comprehensive understanding of water quality and aiding in the identification of pollution sources. This study examined aquatic landscapes at five parks in Xinxiang, China, monitoring thirteen indicators including Water Temperature (WT), Chroma (Ch), Turbidity (Tu), Suspended Solids (SS), Electrical Conductivity (EC), pH, Dissolved Oxygen (DO), Total Nitrogen (TN), Total Phosphorus (TP), Chemical Oxygen Demand (COD), Fe, Zn, and Cu. Utilizing the single-factor evaluation method, the water quality level of each indicator was assessed in accordance with the *Water Quality Standard for Scenery and Recreation Area of the People’s Republic of China* (GB12941-91). The findings revealed significant vertical variations in the levels of TN, TP, COD, Fe, Zn and Cu of aquatic landscapes at parks, while WT, Ch, Tu, SS, EC, and DO showed no marked differences (P>0.05). The monthly dynamics of the water quality indicators indicated generally consistent trends for WT, Ch, Tu, SS, EC, DO, TN, TP, Zn, and Cu, albeit with varying degrees of fluctuation; however, the trends for EC, pH, COD, and Fe exhibited greater variability. These results offer valuable insights for the environmental protection and management of aquatic landscapes in urban parks. Stratified monitoring can capture the dynamic changes in water quality, assisting managers in developing more effective water quality management strategies.

## Introduction

Urban blue-green infrastructure can provide various ecosystem services, such as improving air quality, managing stormwater, offering recreational spaces, and enhancing biodiversity. In recent years, these infrastructures have garnered widespread attention globally [1]. Green spaces and water bodies help mitigate the urban heat island effect, improve air quality, and provide a more comfortable microclimate environment [2]. Numerous studies have shown that green spaces and water bodies not only alleviate stress but also reduce symptoms of depression and anxiety, thereby enhancing overall mental health [3, 4].

Parks serve as a vital component of urban ecological infrastructure and are key to restorative environments [5]. Recreational activities provided by urban parks, such as enjoying tranquility, relaxation, and interaction with nature, are important means of stress relief [6]. They offer scenic views and recreational spaces that enhance mental well-being and alleviate stress. Aquatic landscapes are among the most favored elements in urban parks, frequently incorporated by designers [7]. Ecologically, aquatic landscapes significantly contribute to cooling [8, 9], with the size of the water area being a primary determinant of a park’s cooling capacity [10, 11]. Recreationally, aquatic landscapes heighten public engagement [12], adding to the park’s spatial dynamism [13, 14]. Beyond visual appeal, aquatic landscapes also evoke positive emotions through auditory experiences [15]. Such positive emotions have a direct correlation with the park’s overall size and the average dimension of its aquatic landscapes [16]. Deterioration in water quality significantly reduces the duration of visitors’ stays, whereas areas with better water quality attract more visitors and extend their stay [17].

Water quality stands as a crucial indicator for aquatic landscapes, significantly influencing the duration of tourists’ visits [18]. Aquatic landscape designs frequently offer visitors the chance to engage with water via beaches, creeks, platforms, and the like [19]. Consequently, the water quality in these park aquatic landscapes also bears upon public health. Aquatic landscapes in parks are subject to more stringent water quality standards than those merely for ornamental purposes.。

Except for a few aquatic landscapes that utilize natural aquatic landscapes, the majority of urban parks in China feature artificially constructed aquatic landscapes, predominantly in the form of man-made lakes. These aquatic landscapes often suffer from poor circulation, limited self-cleaning ability, and are susceptible to pollution, particularly during the summer months. Using recycled water to replenish the water is an effective strategy to mitigate water scarcity in urban parks, yet it can also introduce pollution issues [20]. The primary water quality concerns include: The high concentration of total phosphorus in reclaimed water poses a risk of algal blooms [21]. Eutrophication, leading to excessive algae growth, resulting in dark, foul-smelling water that severely impacts the ecological environment [22]; murky water with reduced visibility, detracting from the aesthetic appeal of the landscape; and in some cases, the presence of heavy metals, posing health risks and safety concerns for humans.

Numerous studies have examined the water quality of natural rivers and lakes, yet research on the water quality of small, dispersed aquatic landscapes in urban parks is limited. These studies typically address algal, physical, chemical factors, and spatial configurations. Luo et al. studied the aesthetic preferences and restorative potential of water bodies in ten urban parks in Chengdu [23]. Yin et al. investigated the self-purification model of still ponds in Beijing’s Yuyuantan Park, conducting ecological restoration experiments through various small-scale engineering designs and plant configurations. The results indicated that multi-tiered aquatic plant configurations can effectively inhibit algal blooms [24]. Zhang et al. explored the dynamics of algae in aquatic landscapes in urban parks and their determinants [25]. Water clarity is closely linked to water quality. Chang et al. thus developed a model correlating water transparency with other water quality indicators for assessment purposes [26]. Dou et al. investigated the distribution of heavy metals in urban lakes, suggesting that connectivity projects can enhance water circulation, boost lakes’ self-cleaning abilities, and thus benefit the lakes’ ecological health [27]. Gabr and Soussa (2023) provided a comprehensive evaluation of water quality and its potential uses in the region, focusing on the relationship between surface water quality and the environmental impacts on local communities [28]. Appiah-Opong et al. (2021) explored heavy metal concentrations and the pollution index in drinking water along the southwest coast of Ghana, demonstrating how water quality indicators such as pollution indexes can reflect the contamination levels in water sources used for drinking [29]. Njoku et al. (2024) investigated how water quality can be assessed from a health perspective, which is highly relevant to evaluating water quality in the context of public health [30]. Gabr et al. (2023) provided insights into how urban traffic contributes to pollutants in stormwater runoff, which affects the local environment and infrastructure [31]. Methods for maintaining and restoring river connectivity include system-scale planning, protecting critical freshwater habitats, mitigating the impacts of habitat barriers, and restoring connectivity by removing obstacles and reconnecting rivers, wetlands, and floodplains [32]. The design of the landscape significantly influences water quality, with the adjacent land composition dictating the influx of nutrient-rich substances [33]. Research by Gong et al. indicated that within the three water courses, WT exhibited the most dynamic response, followed by DO and pH levels [34]. For water quality enhancement, fostering extensive underwater plant systems and augmenting the algae-suppressing capacity of reclaimed water in urban landscape ponds are both effective in preventing eutrophication [21, 35]. Restoring aquatic plants can significantly increase water clarity and dissolved oxygen concentration while reducing the concentrations of TN and TP. This restoration also notably enhances the diversity of phytoplankton and reduces the proportion of cyanobacteria [36].

In a closed aquatic system, the absence of material exchange between the upper and lower water courses can readily lead to water quality issues. This study focuses on five park water bodies in Xinxiang City, China, and selects 13 water quality indicators for monitoring. Using the single-factor evaluation method and referencing the "*Water Quality Standard for Scenery and Recreation Area of the People’s Republic of China* (GB12941-91)" the water quality grades for each indicator are determined to assess the overall water quality. To analyze the dynamic changes in water quality, monthly monitoring data were employed to compare water quality indicators, examining the differences between various water layers and their changing trends.

## Materials and methods

### Overview of research area

Xinxiang, situated north of the Yellow River, enjoys a warm temperate continental monsoon climate characterized by four distinct seasons: chilly winters, sweltering summers, crisp autumns, and brisk early springs. The city receives an average annual rainfall of approximately 600mm. Over 70% of the precipitation occurs from June to September, creating a distinct seasonal variation. This precipitation pattern results in frequent heavy rainfall in the summer, which can easily lead to flood disasters, while other seasons remain relatively dry. Its primary natural waterways include the Weihe River, Communist Canal, Mengjiangnu River, and Zhao Dingpai River. These rivers play a crucial role in water diversion, irrigation, and drainage within Xinxiang. The aquatic landscapes in Xinxiang are predominantly found in its larger parks, where they form signature landscapes and provide significant ecological and social benefits.

### Sampling points and time

**Fig 1:**
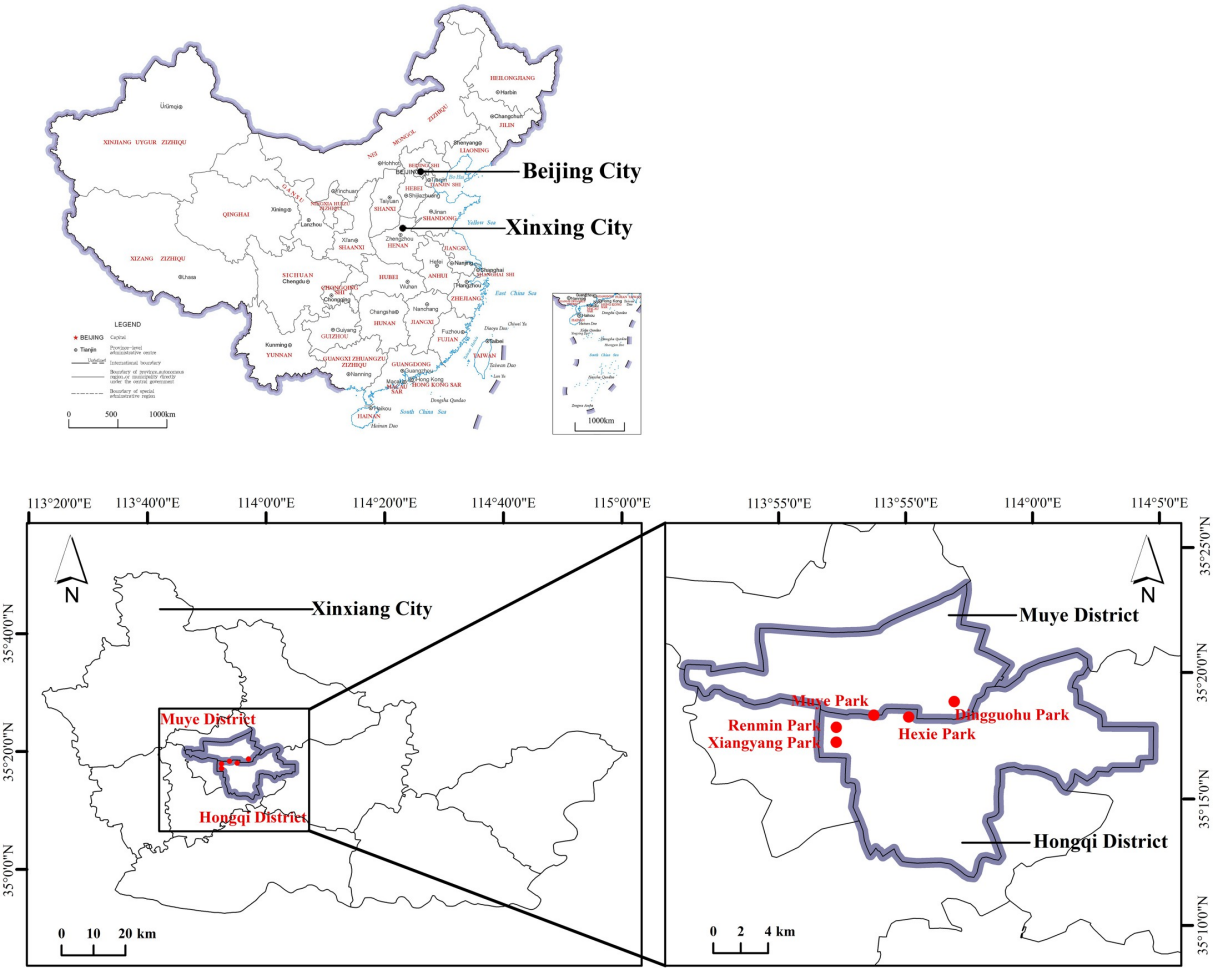
Schematic diagram of park location and distribution (Map sourced from the Ministry of Natural Resources of China, Carto-graphic License: GS (2019)1682).

Xinxiang City has numerous parks, but due to limitations in water resources and construction costs, artificial lakes are mainly concentrated in five larger parks, including Hexie Park, Muye Park, Xiangyang Park, Dingguohu Park, and Renmin Park. The aquatic landscapes within these parks served as the designated sampling areas (Fig 1). Sampling and monitoring were scheduled for the 21st of each month from March to October 2021. Considering safety and engineering constraints, the depth of park landscape water bodies is usually controlled within 2 meters. To ensure successful sampling from all park water bodies, we divided the water bodies into three layers. In each park, three sampling points were established, and handheld samplers were employed to collect water samples at depths of 0.5m, 1.0m, and 1.5m. It is important to note that due to the draining and dredging of the artificial lake in Muye Park by the city government in October, sampling was not successfully conducted. The significant water level fluctuations in People’s Park also led to sampling failures at certain points in some months, but this does not affect the overall water quality assessment.

### Water quality indicators and determination methods

The single-factor evaluation method was employed, whereby the measured values of each indicator were compared with the classification criteria set forth in the national standard to ascertain the water quality level for each parameter. This method clearly illustrates the impact of individual pollutants on water quality, facilitating the rapid identification and assessment of major pollutants. By directly comparing water quality data from different times and locations, it is possible to effectively observe and analyze trends in water quality changes. In accordance with the *Water Quality Standard for Scenery and Recreation Area of the People’s Republic of China (GB12941-91)* (hereafter referred to as the standard), 15 indicators were selected, including WT, Ch, Tu, SS, EC, pH, DO, TN, TP, COD, Fe, Zn, Cu, Cr and Pb. Following two months of monitoring, Cr and Pb were not detected in the water of any park, leading to their exclusion from the analysis, and the focus was maintained on the remaining 13 indicators.

A handheld water sampler was adopted to sample 500ml water samples at each site and store them in brown bottles for subsequent analysis. The Glkrui G968 multi-parameter water quality analyzer was employed to assess the levels of Ch, Tu, and SS. Measurements of WT, EC, pH, and DO were conducted with the Sanxin SX8336 portable water quality analyzer. The concentrations of TP, TN, COD, Cu, Fe, and Zn were determined using the Glkrui G968 multi-parameter water quality analyzer in conjunction with a multi-parameter intelligent digestion instrument. TP levels were measured in accordance with *the GB11893-89 Water Quality-Determination of Total Phosphorus-Ammonium Molybdate Spectrophotometric Method*; TN levels were assessed using *the Water Quality-Determination of Total Nitrogen-Alkaline Potassium Persulfate Digestion-UV Spectrophotometric Method*; COD was determined following *the HJ/T399-2007 Water Quality-Determination Chemical Oxygen Demand-Fast Digestion-Spectrophotometric Method*; Cu was measured via *the Test Methods for Chemical Analysis of Silver Powder for Electric Contact Material-Part 2*: *Determination of Copper Content*; Fe was gauged using *the O-Phenanthroline Spectrophotometric Method; and Zn was quantified by the Xylenol Orange Assay Method*.

### Evaluation method

The water quality levels of each indicator were ascertained by comparing the measured values against the standards. The level reference for indicators in this study is the Water Quality Standard for Scenery and Recreation Area of the People’s Republic of China (GB12941-91). This standard categorizes landscape aquatic landscapes into three classes: Grade A comprises aquatic landscapes in direct contact with the human body; Grade B includes aquatic landscapes in indirect contact with the human body; and Grade C consists of general landscape aquatic landscapes.

### Data processing

Data organization and statistical analysis were performed using Excel 2022 (Developer: Microsoft Corporation; Location: Redmond, Washington, USA) and SPSS 25 (Developer: IBM Corporation; Location: Armonk, New York, USA). One-Way ANOVA was used to compare differences in water quality indicators between different water layers in various parks. Duncan’s Multiple Range Test and Least Significant Difference were applied for multiple comparisons to test the significance of differences between each mean and other means. A P-value of less than 0.05 indicates a significant difference.

### Results and analysis

#### Vertical variation in water quality indicators of aquatic landscapes in different urban parks

**Fig 2:**
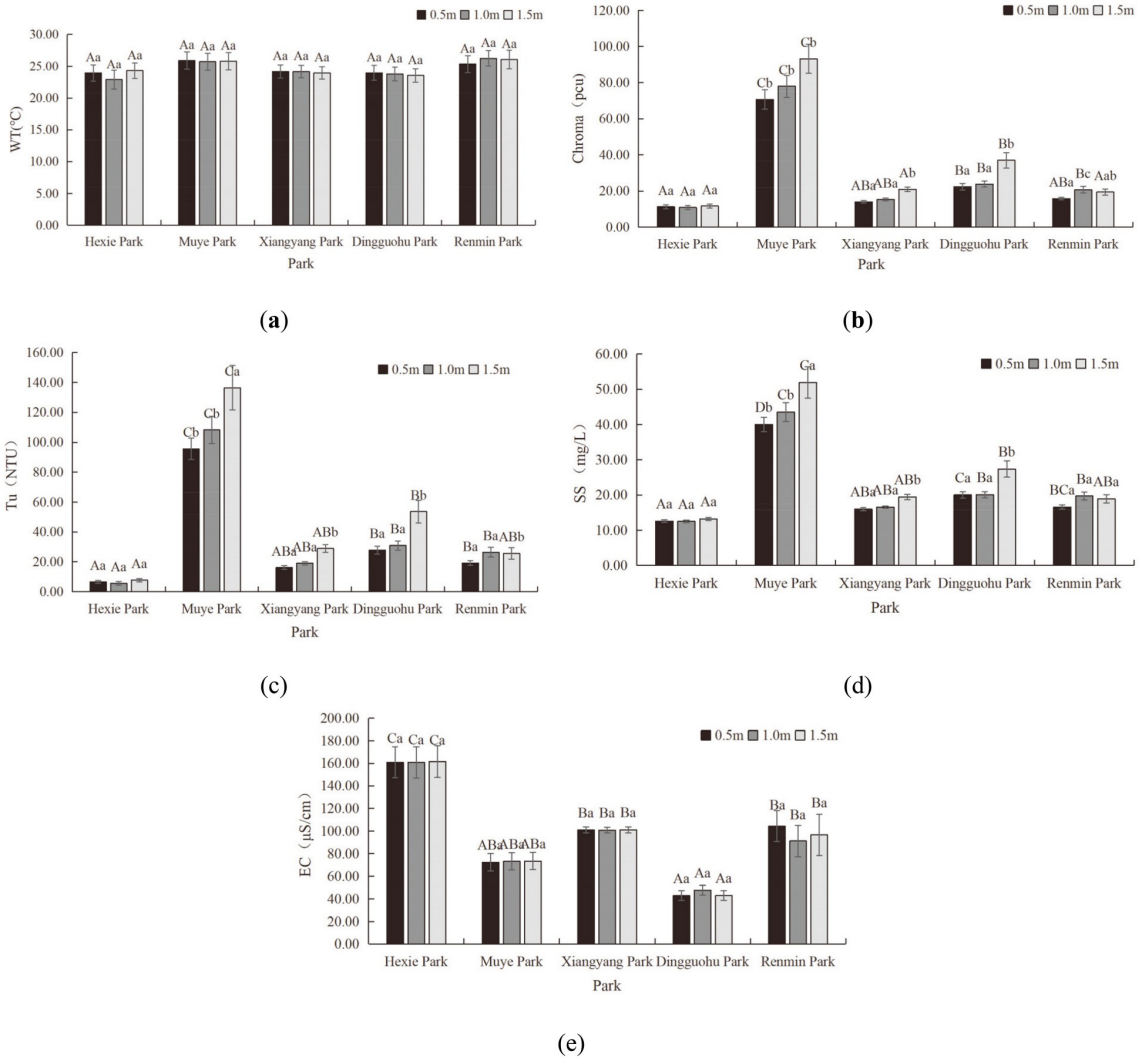
Characteristics of Physical Indicators of aquatic landscapes (WT, Ch, Tu, SS, and EC from a-e, respectively).

*Physical indicator characteristics of aquatic landscapes*. Fig 2 illustrated the vertical variation in physical indicators of the aquatic landscapes.

The vertical variation in WT levels of the aquatic landscapes in different parks was not significant, as followed: 22.9–24.3°C for Hexie Park, 25.79–25.89°C for Muye Park, 23.94–24.17°C for Xiangyang Park, 23.55–23.95°C for Dingguohu Park, 25.34–26.24°C for Renmin Park. Analysis of variance results indicated that the differences in WT of five parks were not statistically significant (P>0.05).

There was no significant vertical difference in Ch levels of aquatic landscapes in Hexie Park. However, the Ch levels of aquatic landscapes at the 1.5m depth in Muye Park, Xiangyang Park, and Dingguohu Park were significantly different from those at the 0.5m and 1.0m depths, with Ch levels increasing with depth. At Renmin Park, the Ch levels of aquatic landscapes at the 1.0m depth were significantly different from those at the 0.5m and 1.5m depths. The Ch levels of aquatic landscapes in different parks at different water courses were as followed: 10.88–11.71pcu for Hexie Park, 70.71–93.19pcu for Muye Park, 14.00–20.96pcu for Xiangyang Park, 22.34–37pcu for Dingguohu Park, 15.84–20.67pcu for Renmin Park. Variance analysis revealed no significant difference in Ch levels between Hexie Park and both Xiangyang Park and Renmin Park (P>0.05), yet a significant difference existed between Hexie Park and Muye Park (P<0.05). Ch levels of Dingguohu Park were not significantly different from those of Xiangyang Park and Renmin Park, but were significantly different from Hexie Park and Muye Park (P<0.05). According to standards, the Ch levels of aquatic landscapes at all water courses in Muye Park and at the 1.5m depth in Dingguohu Park exceed Grade C water quality standards (greater than 25pcu). Exceeding the Ch in water bodies is usually caused by the accumulation of suspended particles, organic matter, and pollutants. These substances not only affect the visual aesthetics of the water but also increase the proliferation of harmful algae and bacteria, endangering aquatic life and public health [37].

In Hexie Park, there was no noticeable vertical difference in Tu levels of aquatic landscapes. However, the Tu levels of aquatic landscapes at the 1.5m depth in Muye Park, Xiangyang Park, Dingguohu Park, and Renmin Park were markedly distinct from the 0.5m and 1.0m depths. In all parks except Renmin Park, the Tu levels tended to increase with the depth of the water course. The Tu level ranges for the different parks were as followed: 5.59–7.76NTU for Hexie Park, 95.62–136.46NTU for Muye Park, 16.19–28.96NTU for Xiangyang Park, 27.85–53.67NTU for Dingguohu Park, and 19.27–26.5NTU for Renmin Park. Variance analysis indicated that the Tu levels in Hexie Park and Xiangyang Park did not significantly differ (P>0.05), but it did significantly differ from other parks (P<0.05). The difference between the Tu levels in Muye Park and other four parks was significant (P<0.05) while the difference between Xiangyang Park and both Dingguohu Park and Renmin Park was not significant (P>0.05).

There was no significant vertical difference in SS levels of aquatic landscapes in Hexie Park and Renmin Park. However, the SS levels of aquatic landscapes at the 1.5m depth in Muye Park, Xiangyang Park, and Dingguo Lake differed significantly from those at the 0.5m and 1.0m depths, with SS levels increasing with depth. The SS levels of aquatic landscapes in different parks were as followed: 12.54–13.21mg/L for Hexie Park, 40.05–51.90mg/L for Muye Park, 16–19.46mg/L for Xiangyang Park, 20.04–27.38mg/L for Dingguohu Park, and 16.58–19.71mg/L for Renmin Park. Variance analysis revealed that there was no significant difference between Hexie Park, Xiangyang Park, and Renmin Park (P>0.05); a significant difference was found between Muye Park and both Hexie Park and Xiangyang Park (P<0.05) yet the difference was not significant between Xiangyang Park, Dingguo Lake and Renmin Park (P>0.05).

No significant vertical differences in EC levels of aquatic landscapes in urban parks were observed. The EC levels of aquatic landscapes in different parks were as followed: 160.86–161.60μS/cm for Hexie Park, 72.41–73.44μS/cm for Muye Park, 100.84–101.1μS/cm for Xiangyang Park, 42.92–47.68μS/cm for Dingguohu Park and 91.29–104.38μS/cm for Renmin Park. Variance analysis results indicated a significant difference in EC levels between Hexie Park and Muye Park, Xiangyang Park, Dingguohu Park, and Renmin Park (P<0.05). The difference in EC levels between Muye Park and Xiangyang Park, Dingguohu Park and Renmin Park was not significant (P>0.05). A significant difference existed between Xiangyang Park and Dingguohu Park (P<0.05), yet a insignificant difference between Xiangyang Park and Renmin Park (P>0.05). Additionally, a significant difference was noted between Dingguohu Park and Renmin Park (P<0.05).

#### Chemical indicator characteristics of aquatic landscapes

**Fig 3:**
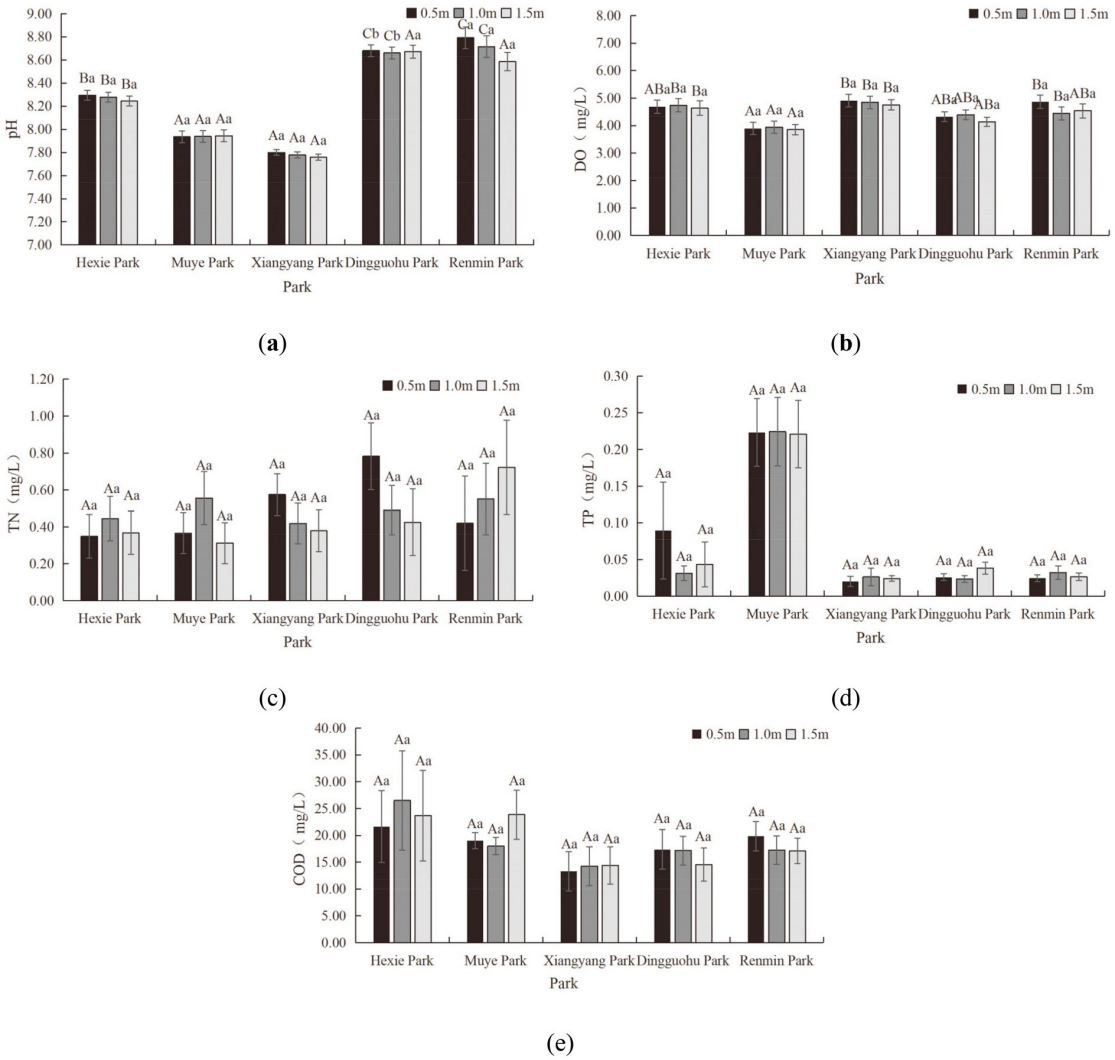
Characteristics of Chemical Indicators (pH, DO, TN, TP and COD from a-e, respectively).

Fig 3 reflected the vertical variation in chemical indicators of the aquatic landscapes.

The pH levels of aquatic landscapes in the parks showed no significant vertical differences. The pH values of aquatic landscapes at different water courses in different parks were as followed: 8.24–8.29 for Hexie Park, 7.94 for Muye Park, 7.76–7.80 for Xiangyang Park, 8.66–8.68 for Dingguohu Park and 8.59–8.79 for Renmin Park. An analysis of variance indicated that the pH levels of aquatic landscapes in five parks did not differ significantly (P>0.05); with the exception of the aquatic landscapes at the depth of 1.5m in Dingguohu Park, which was significantly different from the others, the pH levels of the aquatic landscapes at different water courses in the remaining parks were not significantly distinct (P>0.05). According to standards, the acceptable range for water pH is 6.5–8.5. The water in all parks was alkaline, with the pH levels of aquatic landscapes in Renmin Park and Dingguohu Park exceeding the standard range. Exceeding the pH in water bodies can have widespread negative impacts on aquatic ecosystems, including affecting the growth of aquatic organisms, altering nutrient cycling, and disrupting the biochemical balance of the water. Therefore, monitoring and managing the pH levels of water bodies is crucial [38, 39].

There was no significant vertical difference in DO levels among the aquatic landscapes in the parks. The DO fluctuation ranges of aquatic landscapes at different water courses in different parks were as followed: 4.63–4.74mg/L for Hexie Park, 3.85–3.94mg/L for Muye Park, 4.76–4.92mg/L for Xiangyang Park, 4.14–4.39mg/L for Dingguohu Park and 4.44–4.87mg/L for Renmin Park. The variance analysis revealed that the difference in DO levels in Muye Park and Xiangyang Park was significant (P<0.05). Additionally, the vertical difference in DO levels in Hexie Park, Muye Park, Dingguohu Park and Renmin Park were not significant (P>0.05). According to the standards, all water courses in Xiangyang Park, Renmin Park, Hexie Park, and Dingguohu Park were classified into Grade B (4mg/L≤DO<5mg/L), while all water courses in Muye Park classified into Grade C (3mg/L≤DO<4mg/L). The variation in DO levels in park water bodies is influenced by multiple factors, including organic matter load, algal blooms, water temperature, and water flow. These factors interact, leading to significant fluctuations in DO levels [40, 41].

The TN levels exhibited distinct vertical variations. In Xiangyang Park and Dingguohu Park, TN decreased with increasing water depth. Conversely, in Renmin Park, TN rose with greater depth. The TN levels of the aquatic landscapes at the depth of 1.0m in Hexie Park and Muye Park were markedly higher than those at other depths. The TN fluctuation range of aquatic landscapes at different water courses in different parks were as followed: 0.35–0.45mg/L for Hexie Park, 0.31–0.56mg/L for Muye Park, 0.38–0.57mg/L for Xiangyang Park, 0.43–0.78mg/L for Dingguohu Park, and 0.42–0.72mg/L for Renmin Park. Variance analysis indicated no significant differences in TN levels between parks (P>0.05).

The vertical difference in the TP levels was distinct. With the exception of Hexie Park and Dingguohu Park, where the TP at the depth of 1.0m was lower than at 0.5m and 1.5m, the TP levels at the depth of 1.0m in other parks surpassed that at the depth of 0.5m and 1.5m. The TP fluctuation range of aquatic landscapes at different water courses in different parks were as followed: 0.03–0.09mg/L for Hexie Park, Muye Park consistently at 0.22 mg/L for all water courses, 0.02–0.03mg/L for Xiangyang Park, 0.02–0.04mg/L for Dingguohu Park, and 0.025–0.03mg/L for Renmin Park. Variance analysis results indicated that the TP levels in Muye Park were significantly higher than in other four parks (P<0.05). Based on the standards, the water in Xiangyang Park, Dingguohu Park, and Renmin Park all qualified as Grade C water (0.02mg/L<TP≤0.05mg/L) while water in Hexie Park and Muye Park classified into above Grade C water (TP>0.05mg/L). These high concentrations of nutrients can lead to eutrophication and excessive algal growth, which adversely affect the growth of aquatic plants and animals, thereby disrupting the balance of the aquatic ecosystem [42].

The COD levels of aquatic landscapes in the parks exhibited distinct vertical variations. In Dingguohu Park and Renmin Park, the COD at the depth of 0.5m was higher than at the depth of 1.0m and 1.5m. Conversely, the water at the depth of 0.5m in Hexie Park and Xiangyang Park had lower COD levels than at the depth of m and 1.5m. The COD levels at the depth of 1.5m was the highest. The COD fluctuation range of different water courses in different parks were as followed: 21.63–26.5mg/L for Hexie Park, 18–23.86mg/L for Muye Park, 13.3–14.38mg/L for Xiangyang Park, 14.54–17.38mg/L for Dingguohu Park, and 17.07–19.83mg/L for Renmin Park. Variance analysis indicated that the vertical differences in COD levels among the five parks were not statistically significant (P>0.05). High COD values are significantly associated with organic pollution in water bodies, leading to reduced water clarity and DO content, which significantly impacts the health and survival of aquatic organisms, particularly fish [43, 44].

#### Heavy metal indicator characteristics of aquatic landscapes

**Fig 4:**
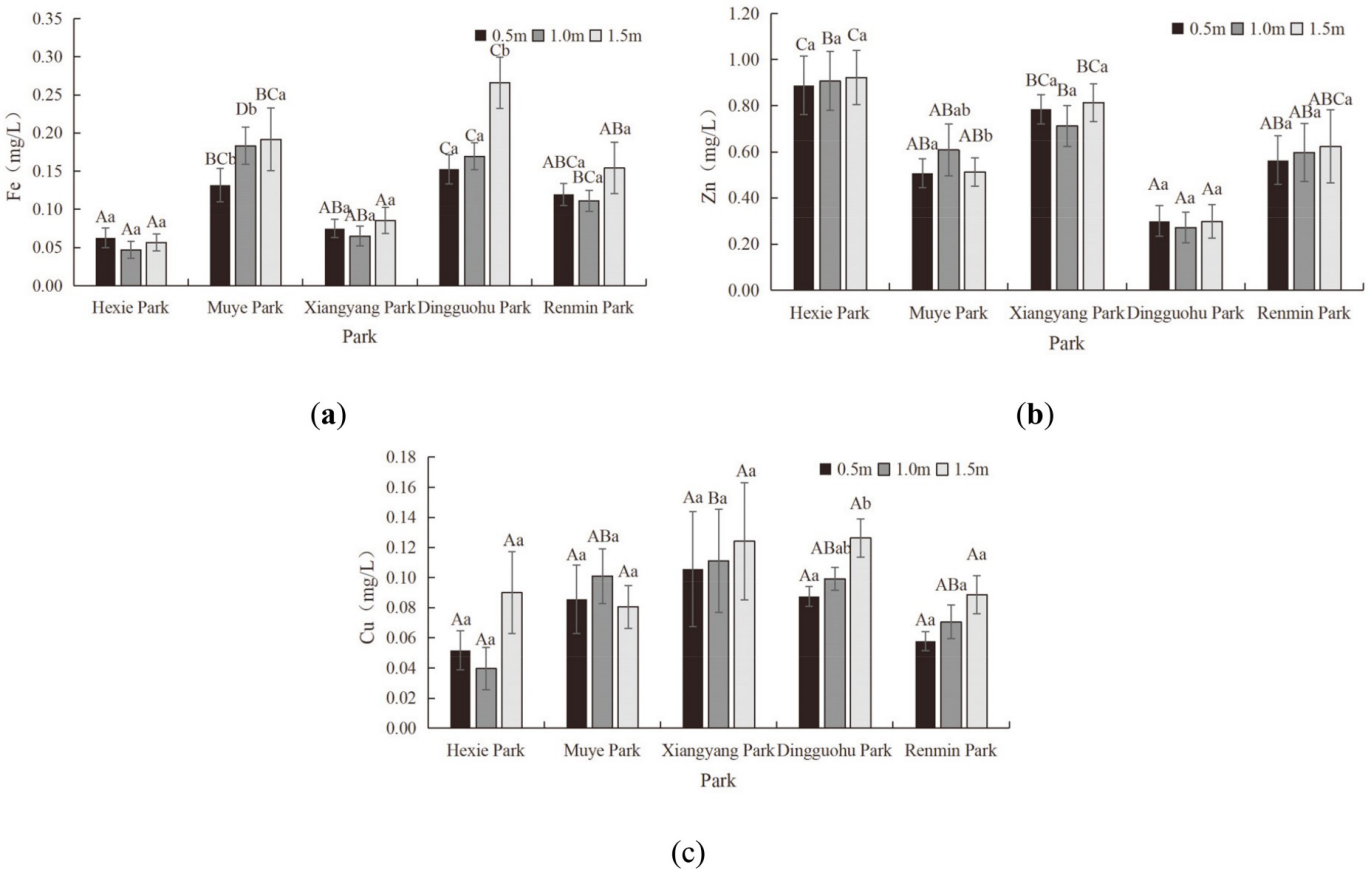
Characteristics of Heavy Metal Indicators (Fe, Zn and Cu from a-c, respectively).

Fig 4 illustrated the vertical variation in heavy metal indicators of the aquatic landscapes.

The Fe content in the water courses of various parks exhibited distinct variations. In Hexie Park, Xiangyang Park, and Renmin Park, the water course at the depth of 1.0m had higher Fe content than the depth of 0.5m and 1.5m. Conversely, the water course at the depth of 1.5m in Muye Park and Dingguohu Park contained more Fe than at the depth of 0.5m and 1.0m. The range of Fe content of different water courses in different parks were as followed: 0.047–0.06mg/L for Hexie Park, 0.13–0.19mg/L for Muye Park, 0.07–0.09mg/L for Xiangyang Park, 0.15–0.27mg/L for Dingguohu Park, and 0.11–0.15mg/L for Renmin Park. Variance analysis revealed that there was no significant difference in Fe content between Hexie Park, Xiangyang Park, and Renmin Park (P>0.05). However, a significant difference existed between Muye Park and Dingguohu Park (P<0.05). The difference between Muye Park and Dingguohu Park, Dingguohu Parkn and Renmin Park was not significant (P>0.05). There was a significant difference between Xiangyang Park and Dingguohu Park (P<0.05), yet an insignificant difference with Renmin Park (P>0.05). Similarly, Dingguohu Park and Renmin Park did not differ significantly (P>0.05). According to standards, the water in all five parks were classified as Grade A water (Fe≤0.3mg/L). Increased Fe concentrations can cause water turbidity, thereby affecting the survival of aquatic plants and animals [45].

The vertical difference in Zn content was distinct. In Hexie Park and Renmin Park, the Zn content increased with depth. With the exception of the water course at the depth of 1.0m in Muye Park, where the concentration was higher than at 0.5m and 1.5m, the Zn content at the depth of 1.0m in Xiangyang Park and Dingguohu Park was lower than at the depth of 0.5m and 1.5m. The range of Zn content of different water courses in different parks were as followed: 0.89–0.92mg/L for Hexie Park, 0.51–0.61mg/L for Muye Park, 0.71–0.81mg/L for Xiangyang Park, 0.27–0.30mg/L for Dingguohu Park, and 0.56–0.62mg/L for Renmin Park. Variance analysis indicated that there was no significant difference in the Zn content between Hexie Park and Muye Park, Xiangyang Park, or Renmin Park (P>0.05), but there was a significant difference between Hexie Park and Dingguohu Park (P<0.05). The difference between Muye Park and Xiangyang Park, Dingguohu Park and Renmin Park was significant (P>0.05). Xiangyang Park and Dingguohu Park showed a significant difference (P<0.05), yet an insignificant difference with Renmin Park (P>0.05). Similarly, Dingguohu Park and Renmin Park showed no significant difference (P>0.05). According to the standards, the water in all five parks was classified as Grade C (0.1mg/L<Zn≤1.0mg/L). Excess Zn in park water systems can disrupt ecological balance, harm aquatic life, and degrade water quality. To protect these ecosystems from Zn pollution, the implementation of effective management and mitigation strategies is crucial [46].

The Cu content in different water courses exhibited distinct variations. In Xiangyang Park, Dingguohu Park, and Renmin Park, the Cu content increased with water depth. At Hexie Park, the Cu content at the depth of 1.0m was lower than at the depth of 0.5m and 1.5m, whereas at Muye Park, it was higher at the depth of 1.0m compared to 0.5m and 1.5m. The range of Cu content of different water courses in different parks were as followed: 0.04–0.09mg/L for Hexie Park, 0.08–0.10mg/L for Muye Park, 0.11–0.12mg/L for Xiangyang Park, 0.09–0.13mg/L for Dingguohu Park, and 0.06–0.09mg/L for Renmin Park. Variance analysis indicated that the differences in Cu content across the five parks were not statistically significant (P>0.05). Based on the standards, the water in Hexie Park, Muye Park, and Renmin Park was classified as Grade C water (0.01mg/L<Cu≤0.1mg/L). All water courses in Xiangyang Park and the water course at the depth of 1.5m in Dingguohu Park exceeded the threshold for Grade C water. Cu is significantly toxic to various aquatic organisms. Elevated Cu levels can reduce water clarity and impact overall water quality health. High concentrations of Cu not only affect the growth and reproduction of aquatic life but also lead to ecological imbalance [47, 48].

### Dynamic changes in water quality indicators of different parks

#### Dynamic changes in physical indicators of aquatic landscapes

**Fig 5:**
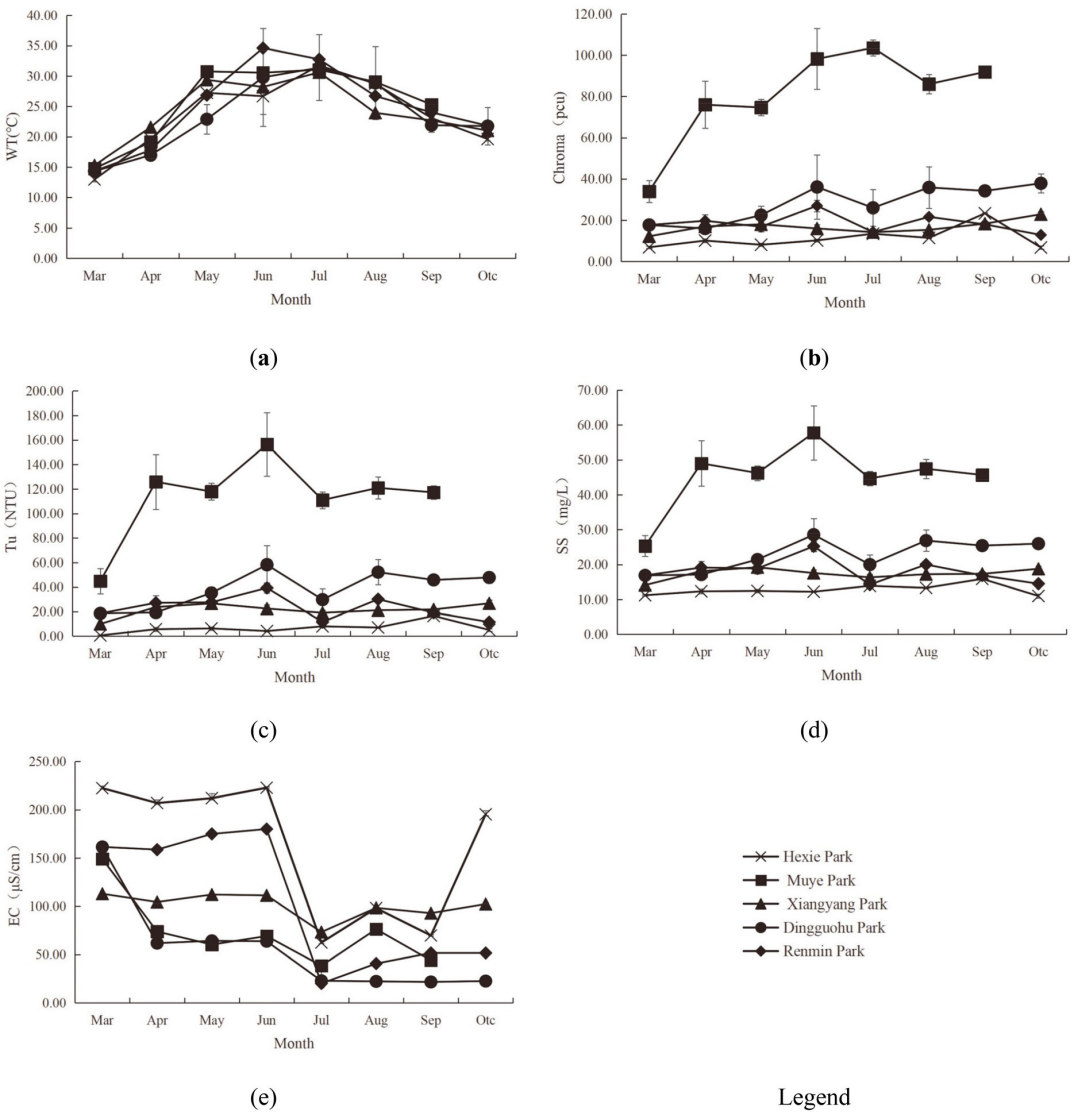
Dynamic Changes in Physical Indicators (Monthly Variations in WT, Ch, Tu, SS and EC from a-e, respectively).

Fig 5 illustrated the monthly fluctuations in the water quality indicators.

The monthly WT ranges for park aquatic landscapes were as followed: 12.98–31.74°C for Hexie Park, 14.78–31.04°C for Muye Park, 15.30–30.60°C for Xiangyang Park, 14.35–31.38°C for Dingguohu Park, and 14.35–34.0°C for Renmin Park. The monthly dynamic trends of WT across these parks were generally consistent, with temperatures rising gradually until peaking in June and July, followed by a steady decline.

The monthly Ch ranges for park aquatic landscapes were as followed: 6.89–23.33pcu for Hexie Park, 34.0–103.56pcu for Muye Park, 12.22–22.89pcu for Xiangyang Park, 16.0–37.89pcu for Dingguohu Park, and 12.88–27.0pcu for Renmin Park. The monthly dynamic trends of Ch across the parks were generally consistent, exhibiting a gradual upward trend. Notably, Muye Park experienced significant month-to-month fluctuations in Ch, while the other parks showed more stable variations. According to the standards, the water in Hexie Park, Xiangyang Park, and Dingguohu Park was classified as Grade C (less than 25pcu) from March to May; the water in Muye Park exceeded Grade C (more than 25pcu) every month; and the water in Renmin Park was rated as Grade C, with the exception of June when the water surpassed the Grade C standard.

The monthly Tu ranges for park aquatic landscapes were as followed: 0.60–16.51NTU for Hexie Park, 44.96–156.33NTU for Muye Park, 9.97–26.71 NTU for Xiangyang Park, 18.51–58.26NTU for Dingguohu Park, and 11.54–39.31NTU for Renmin Park. The monthly dynamic trends for each park’s aquatic landscapes were generally consistent, exhibiting a gradual increase. Notably, Muye Park’s monthly variations showed significant fluctuations, whereas the other parks demonstrated more stable trends.

The monthly SS ranges for park aquatic landscapes were as followed: 11.0–15.89mg/L for Hexie Park, 25.33–57.78mg/L for Muye Park, 14.11–19.22mg/L for Xiangyang Park, 16.89–28.56mg/L for Dingguohu Park, and 14.25–25.25mg/L for Renmin Park. The monthly dynamic trend of SS across the parks was generally consistent, exhibiting a gradual increase. Notably, Muye Park experienced significant monthly SS fluctuations, whereas the other parks demonstrated more modest variations.

The monthly EC ranges for park aquatic landscapes were as followed: 62.46–222.67μS/cm for Hexie Park, 38.48–149.0μS/cm for Muye Park, 73.33–112.96μS/cm for Xiangyang Park, 21.72–161.39μS/cm for Dingguohu Park, and 20.15–179.93μS/cm for Renmin Park. The EC changes of Muye Park and Dingguohu Park showed a downward trend month by month. The EC changes of Xiangyang Park did not fluctuate significantly from month to month. The EC changes of Hexie Park and Renmin Park showed little fluctuation from March to June. They began to decline sharply in June, and then rose gradually.

#### Dynamic changes in water chemical indicators

**Fig 6:**
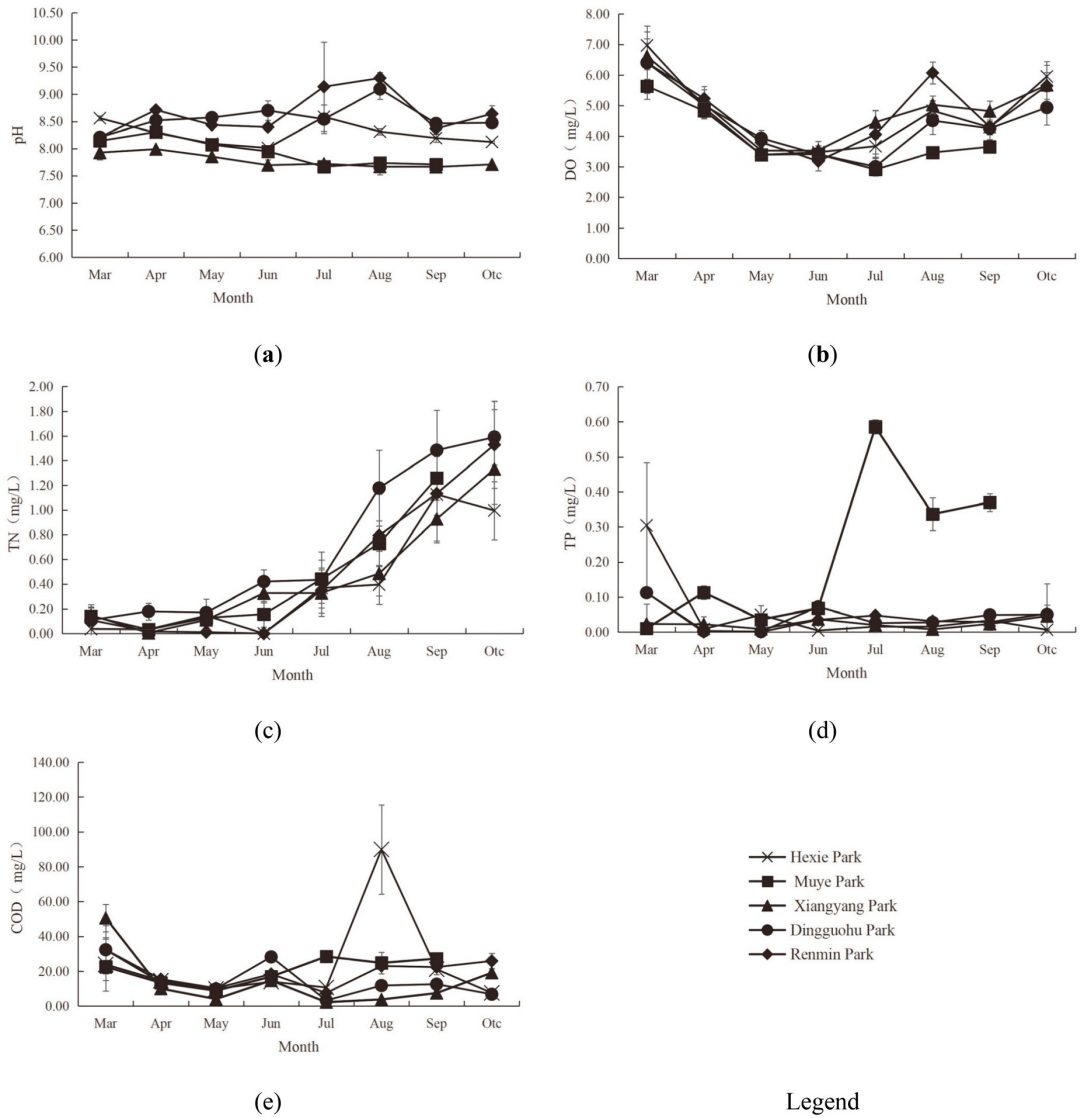
Dynamic Changes in Chemical Indicators (Monthly Variations in pH, DO, TN, TP and COD from a-e, respectively).

Fig 6 showed the monthly dynamics in the chemical indicators.

The monthly pH ranges for park aquatic landscapes were as followed: 8.01–8.59 for Hexie Park, 7.67–8.31 for Muye Park, 7.66–8.00 for Xiangyang Park, 8.21–9.10 for Dingguohu Park, and 8.21–9.30 for Renmin Park. The monthly pH changes in Dingguohu Park and Renmin Park were roughly the same, rising first and then falling, reaching a peak in August. The pH of other parks did not fluctuate significantly from month to month. According to the standards, the pH values of Muye Park every and Xiangyang Park all the year around, Hexie Park from April to June and from August to October, Dingguohu Park in March, May, June, September and October were normal (6.5<pH<8.5) while the pH indicators of Hexie Park in March and July, Dingguohu Park in April, May, June, July and August, and Renmin Park in April, July, August and October exceeded the standard range (pH>8.5).

The monthly DO ranges for park aquatic landscapes were as followed: 3.39–6.97mg/L for Hexie Park, 2.91–5.63mg/L for Muye Park, 3.52–6.61mg/L for Xiangyang Park, 3.00–6.40mg/L for Dingguohu Park, and 3.18–6.40mg/L for Renmin Park. The monthly DO trend in each park was generally consistent, with levels gradually decreasing until June and July, then rising again. Based on the standards, the water quality of Hexie Park was rated as Grade A (DO≥5 mg/L) in March and October, Grade B (4mg/L≤DO<5mg/L) in April, August, and September, and Grade C (3mg/L≤DO<4mg/L) in May, June, and July. Muye Park had Grade A water in March, Grade B in April, and Grade C in May, June, August, and September. The water of Xiangyang Park was rated as Grade A in March, April, August and October, Grade B in July and September, and Grade C in May and June. Dingguohu Park had Grade A water in March and April, Grade B in August, September, and October, and Grade C from May to July. Renmin Park featured Grade A water in March, April, August, and October, Grade B in July and September, and Grade C in May and June.

The monthly TN ranges for park aquatic landscapes were as followed: 0.00–0.10mg/L for Hexie Park, 0.03–0.73mg/L for Muye Park, 0.01–0.93mg/L for Xiangyang Park, 0.11–1.60mg/L for Dingguohu Park, and 0.00–1.53mg/L for Renmin Park. The monthly TN trend in each park was generally consistent, exhibiting a month-over-month increase. The variations from March to June were relatively minor, whereas those from June to October were more pronounced.

The monthly TP ranges for park aquatic landscapes were as followed: 0.004–0.30mg/L for Hexie Park, 0.01–0.59mg/L for Muye Park, 0.01–0.05mg/L for Xiangyang Park, 0.001–0.10mg/L for Dingguohu Park, and 0.00–0.10mg/L for Renmin Park. With the exception of Muye Park, the monthly TP trends in the other parks were relatively stable with minimal fluctuation. Muye Park, however, exhibited a pattern of increasing to a peak in July and then gradually declining. According to the standards, Hexie Park’s water quality was rated as Grade A and B (TP≤0.02 mg/L) in April, June, July, August, and October, and Grade C in Mary and September (0.02mg/L<TP≤0.05mg/L) and below Grade C (TP>0.05mg/L) in March. Muye Park had Grade A and B water in March, Grade C in May, and below Grade C in April, June, July, August, and September. The water of Xiangyang Park met Grade A and B criteria in May, July, and August, and Grade C in March, April, June, September, and October. Dingguohu Park had Grade A and B water in April and May, Grade C water from July to September, and below Grade C in March, June, and October. Renmin Park had Grade A and B water in April and May, Grade C from June to September, and blow Grade C in March and October.

The monthly COD ranges for park aquatic landscapes were as followed: 7.56–89.89mg/L for Hexie Park, 0.01–0.59mg/L for Muye Park, 8.78–28.44mg/L for Xiangyang Park, 2.22–50.56mg/L for Dingguohu Park, and 3.22–32.33mg/L for Renmin Park. The overall monthly COD variations across the parks were quite pronounced. Hexie Park’s COD levels initially rose, peaking in August before declining; Muye Park, Dingguohu Park, and Renmin Park each exhibited an initial decrease, followed by a gradual increase and then stabilization; Xiangyang Park’s COD trended downward slowly before rising as the months progressed.

#### Dynamic changes in heavy metal indicators

**Fig 7:**
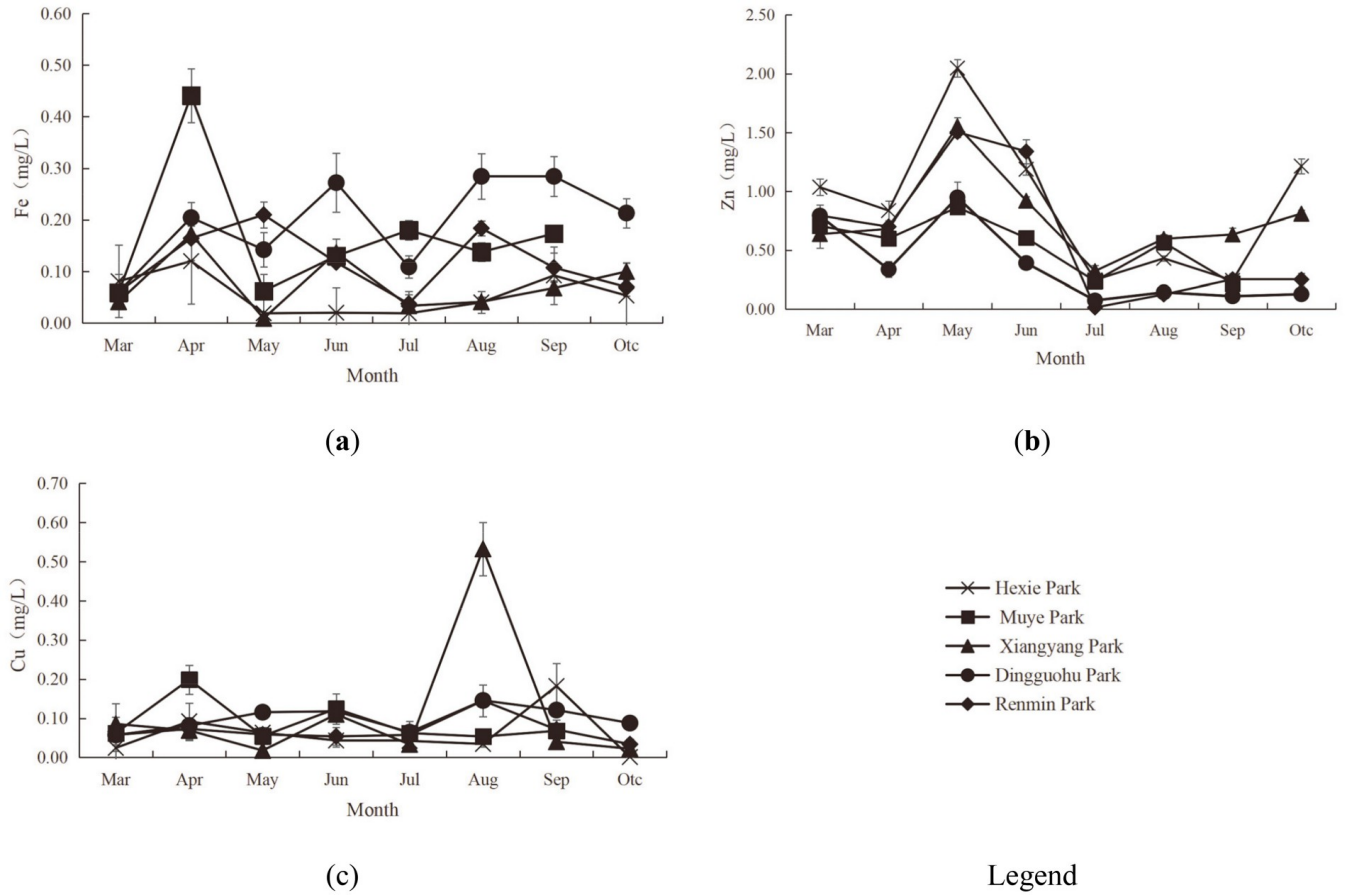
Dynamic Changes in Heavy Metal Indicators (Monthly Variations in Fe, Zn and Cu from a-c, respectively).

Fig 7 illustrated the monthly dynamics of heavy metal indicators.

The monthly Fe ranges for park aquatic landscapes were as followed: 0.019–0.12mg/L for Hexie Park, 0.06–0.44mg/L for Muye Park, 0.01–0.17mg/L for Xiangyang Park, 0.06–0.28mg/L for Dingguohu Park, and 0.04–0.21mg/L for Renmin Park. The monthly changes and fluctuations of the Fe levels in Hexie Park were subtle. Muye Park peaked in April, followed by a gradual decline. Xiangyang Park’s Fe levels were higher in April, then decreased before a slight increase. Dingguohu Park showed a steady rise until June, then a gradual decline, with minimal fluctuations from August to October. The Fe indicator of Renmin Park reached its highest in May and August. According to standards, the water of Hexie Park, Xiangyang Park, Dingguohu Park, and Renmin Park qualified as Grade A water (Fe≤0.3 mg/L) from March to October while that of Muye Park as Grade B water (0.3mg/L<Fe≤0.5mg/L) in April and Grade A water in rest months.

The monthly Zn ranges for park aquatic landscapes were as followed: 0.24–2.05mg/L for Hexie Park, 0.22–0.86mg/L for Muye Park, 0.32–1.55mg/L for Xiangyang Park, 0.07–0.95mg/L for Dingguohu Park, and 0.01–1.50mg/L for Renmin Park. The monthly trends of Zn levels in each park were largely in sync, typically peaking in May before gradually decreasing. Hexie Park experienced the most significant month-to-month changes, while Muye Park and Dingguohu Park exhibited smaller fluctuations. Xiangyang Park and Renmin Park displayed similar fluctuation patterns. According to the standards, from March to October, both the water of Muye Park and Dingguohu Park qualified as Grade C water (Zn≤1.0mg/L). The water of Hexie Park met Grade C criteria in April, July, August, and September. The water of Xiangyang Park was rated as Grade C in all months except May. In months other than May, Xiangyang Park’s Zn levels exceeded the Grade C threshold (Zn>1.0mg/L). The water of Renmin Park was rated as Grade C in May and June, as below Grade C in all other months.

The monthly Cu ranges for park aquatic landscapes were as followed: 0.001–0.1mg/L for Hexie Park, 0.05–0.20mg/L for Muye Park, 0.02–0.53mg/L for Xiangyang Park, 0.06–0.15mg/L for Dingguohu Park, and 0.03–0.15mg/L for Renmin Park. Except for Xiangyang Park, where the Cu indicator was relatively high in August, the monthly change trends of the other parks were generally consistent, with no obvious fluctuations. According to the standards, Hexie Park had Grade A and B water (Cu≤0.01mg/L) in October, Grade C water from March to August (0.01mg/L<Cu≤0.1mg/L), and water below Grade C in September (Cu>0.1mg/L). Muye Park had Grade C water in March, May, July and September, water below Grade C in April and June. The water quality of Xiangyang Park was rated as Grade C from March to May, July, September and October, below Grade C in August. The water of Dingguohu Park qualified as Grade C water in March, April, July and October, water below Grade C in May, June, August and September. Renmin Park had Grade C water from March to July, September and October while the water was below Grade C in August.

## Discussion

Under typical conditions, water pollution primarily originates from diffuse non-point sources linked to nearby residential and industrial activities. Urban stormwater runoff can carry pollutants into water bodies, and sometimes residential and commercial wastewater is also directly or indirectly discharged into park water bodies [49, 50]. Many parks suffer from issues like limited water volumes, stagnant flow, and diminished self-cleaning ability. The slow renewal of aquatic landscapes can readily lead to water quality concerns [51]. In urban parks, the main sources of water supply are reclaimed water, municipal tap water, and rainwater. The introduction of water with higher levels of pollutants exacerbates the contamination of aquatic landscapes.

Aquatic landscapes such as large lakes and reservoirs are typically deep and stratified [52]. The surface WT of reservoirs is relatively high, while the bottom WT is lower. This thermal stratification limits the vertical mixing of oxygen, leading to oxygen deficiency in the bottom water and the formation of hypoxic conditions [53]. During thermal stratification, the concentration of phytoplankton is higher in the surface water and lower in the bottom water [54]. The stratification of park water quality is primarily due to differences in temperature, chemical composition, and biological activity at different water layers. For instance, thermal stratification of the water body is particularly pronounced during the summer [55]. However, most aquatic landscapes at the urban parks in Xinxiang are shallow, with depths not exceeding 2m. In the absence of flowing water sources, disturbances such as wind, rainwater inflow, and recreational activities can cause material exchange between water courses [56]. However, the intensity, flow, and frequency of these phenomena are irregular, and their occurrence locations and ranges are uncertain, which increases the difficulty of analyzing water quality stratification. Consequently, the stratification of physical water quality indicators is not pronounced. These indicators, primarily visual, impact the aesthetic quality of aquatic landscapes. In contrast, the stratification of chemical and heavy metal indicators is more evident, though the patterns vary. Due to the complexity of their formation, a thorough investigation upon the surrounding environment is of great necessity for a comprehension understanding. Specifically, the chemical quality of the shallow water course at Hexie Park was subpar, and the TN levels of the shallow water courses at Xiangyang Park and Dingguohu Park were elevated. This could be attributed to pollution from surface runoff, with rainwater carrying nutrients that increase TN levels in the shallow water courses [57]. Nutrient and organic matter-rich eroded soil is also a significant source of water pollution. In the deeper water courses at Muye Park, Xiangyang Park, and Dingguohu Park, higher levels of Ch, Tu and SS were observed. Muye Park’s water courses exhibited lower DO levels, and the deep water course had the highest COD levels. The poor physical quality of the deep water course might result from reduced transparency due to sediment pollution [58]. Additionally, the deep water courses in most parks contain high concentrations of heavy metals, likely influenced by the release from sediments [59].

According to the monthly fluctuations of aquatic conditions across various parks, it is evident that the majority of indicators exhibit similar trends, with only a select few showing significant variances. This indicates that the entire water body is affected by the same pollution sources or environmental factors, allowing for the implementation of unified management and control strategies to address water quality issues, thereby improving management efficiency [60]. This requires controlling pollution at the source, such as reducing agricultural runoff, industrial wastewater discharge, and pollutants from urban runoff [61]. Over 70% of Xinxiang City’s precipitation occurs from June to September. Generally, during the rainy season in summer, most parks experience a decline in water quality due to chemical indicators. This deterioration is largely attributed to the influx of nitrogen, phosphorus, and other pollutants carried by rainwater and surface runoff into the aquatic landscapes [62]. This aligns with the findings on the hydrochemical characteristics of the Qingshui River in Guizhou during the wet season [63]. Rainfall and runoff in urban areas can carry large amounts of pollutants, such as organic matter, heavy metals into park water bodies [64]. During this period, the nutrient-rich conditions promote algae growth, often leading to visible blue-green algae blooms. The data also indicate that concentrations of various metallic elements tend to be higher during wet seasons compared to dry ones. These findings resonate with the research on the chemical composition and spatial distribution of heavy metals in the Haihe River’s main waterway [65]. Specifically, the Zn levels in each park peaked in May, with Xiangyang Park and Renmin Park showing elevated Zn concentrations. Given their proximity to residential areas and thoroughfares, it is likely that the primary contributors to these levels are household wastewater and vehicle emissions. Of course, the specific causes require a more detailed investigation of the surrounding environment of the park.

Increased water body flow can effectively improve water quality by reducing the accumulation of pollutants [66]. In managing water quality within park aquatic landscapes, it’s crucial to prioritize human health. Methods such as physical barriers, pre-treatment of water, and ecological management should be primarily employed. Physical barriers involve the use of stormwater interception infrastructure to prevent surface runoff, particularly from roads, from entering the aquatic landscapes. This is effective in reducing the influx of nutrients and heavy metals like nitrogen and phosphorus [56]. Pre-treatment of water focuses on the rainwater from surrounding green spaces, utilizing extensive stormwater systems to adsorb nutrients and heavy metals on-site, thereby lessening their discharge into the aquatic landscapes [67]. Ecological management includes the introduction of disturbance mechanisms or the cultivation of aquatic plants. Aeration devices significantly raise the oxygen levels, encourage inter-course circulation, and markedly enhance the physical indicators [68]. The implementation of extensive underwater plant systems and wetland vegetation not only absorbs chemical substances and heavy metals but also fosters a healthy aquatic ecosystem [69, 70]. In addition, Hybrid-integrated-novel approaches/techniques, which combine surface flow constructed wetlands, aeration, ecological gravel beds, and wetland multi-pond systems, can effectively remove pollutants and improve water quality [71].

## Conclusion

Artificial lakes, as important blue infrastructure in cities, play a crucial role in improving the urban ecological environment and providing high-quality recreational spaces. However, these water bodies vary in shape, are relatively enclosed, and lack water flow. Additionally, being situated in complex urban environments, they are prone to disturbances from human activities, resulting in water quality characteristics that differ from natural lakes. Indicator analysis based on stratified sampling and inter-monthly dynamic monitoring helps to identify the patterns of water quality changes, providing a basis for the formulation of targeted management strategies.

There are marked vertical differences in the TN, TP, COD, Fe, Zn, and Cu levels. In terms of the monthly dynamics in the water quality indicators, the trends for WT, Ch, Tu, SS, DO, TN, TP, Zn and Cu are broadly aligned, albeit with varying degrees of fluctuation; however, the trends for EC, pH, COD and Fe diverge significantly. Factors such as surface runoff from rainfall, sediment contamination, and domestic wastewater are the primary contributors. Considering that landscape water bodies may come into contact with humans, chemical methods are not suitable. Strategies such as physical barriers, pre-treatment of water, ecological management practices, and Hybrid-integrated-novel approaches should be considered for remediation.

## Supporting information

S1 File (XLSX)
